# Anti‐MAG Polyneuropathy: Characterization of the Monoclonal Gammopathy and Clonal B‐Cell Population

**DOI:** 10.1155/jimr/1959235

**Published:** 2026-07-03

**Authors:** Geir Erland Tjønnfjord, Liv Toril N. Osnes, Signe Spetalen, Ulla Haave, Trine H. Popperud, Angelina H. Maniaol, Ingunn Østlie, Livia Bajelan, Lars Henrik D. Hamnvik, Kristo Marvyin, Dragana Miskovic, Eirik B. Tjønnfjord, Kristoffer Winje, Magnus Moksnes, Tor Henrik A. Tvedt, Ann Kristin Kvam, Agnieszka Malecka

**Affiliations:** ^1^ Department of Haematology, Oslo University Hospital, Oslo, Norway, oslo-universitetssykehus.no; ^2^ Institute of Clinical Medicine, University of Oslo, Oslo, Norway, uio.no; ^3^ Department of Immunology, Oslo University Hospital, Oslo, Norway, oslo-universitetssykehus.no; ^4^ Department of Pathology, Oslo University Hospital, Oslo, Norway, oslo-universitetssykehus.no; ^5^ Department of Pathology, Akershus University Hospital, Lørenskog, Norway, ahus.no; ^6^ Department of Neurology, Oslo University Hospital, Oslo, Norway, oslo-universitetssykehus.no; ^7^ Department of Internal Medicine, Vestre Viken Hospital Trust, Drammen, Norway, vestreviken.no; ^8^ Department of Hematology, Akershus University Hospital, Lørenskog, Norway, ahus.no; ^9^ Department of Internal Medicine, Telemark Hospital Trust, Skien, Norway, sthf.no; ^10^ Department of Internal Medicine, Østfold Hospital Trust, Grålum, Norway, sykehuset-ostfold.no; ^11^ Department of Internal Medicine, Innlandet Hospital Trust, Gjøvik, Norway, sykehuset-innlandet.no; ^12^ Cancer and Hematology Center, Vestfold Hospital Trust, Tønsberg, Norway, siv.no

## Abstract

Polyneuropathy due to antibodies to myelin‐associated glycoprotein (MAG) is a rare disease with an estimated prevalence of 1 per 100,000. Symptoms start in the sixth or seventh decade of life, presenting sensory polyneuropathy with sensory ataxia, paresthesia, mild motor deficit, and tremor of upper extremities. The neurophysiological features are compatible with length‐dependent demyelination. It is caused by monoclonal gammopathy of the IgM type produced by clonal B cells. In recent years the monoclonal anti‐CD20 antibody has become the preferred first‐line treatment, but with limited efficacy. We identified a cohort of 42 patients diagnosed with anti‐MAG polyneuropathy. Clinical data were collected retrospectively, and flow cytometry files and immune histochemistry slides were reassessed. We report that in most cases of anti‐MAG polyneuropathy, the monoclonal IgM is kappa‐restricted, the B‐cell population shows lymphoplasmacytic differentiation, MYD88^L265P^ mutation is a frequent finding, and the clonal B‐cell population includes plasma cells. These findings most likely explain the low efficacy of rituximab monotherapy. We found the burden of symptoms to be high among patients with anti‐MAG polyneuropathy as most of our patients had received intravenous immunoglobulin infusions and/or B‐cell‐directed treatment. We suggest that future treatment protocols for anti‐MAG polyneuropathy should incorporate plasma cell‐directed drugs. Furthermore, we found complement receptor 1 (CD35) to be down regulated on clonal B lymphocytes but not on normal B lymphocytes in these patients. We suggest that this may be a common feature of clonal B lymphocytes in chronic lymphoproliferative diseases.

## 1. Introduction

In patients with polyneuropathy and IgM monoclonal gammopathy, the IgM antibodies show reactivity to several neural targets, myelin‐associated glycoprotein (MAG) in particular [1, 2]. Anti‐MAG polyneuropathy is a rare disease with an estimated prevalence of 1 per 100 000 [2, 3]. The disease typically begins in the sixth or seventh decade of life as a slowly progressive distal sensory polyneuropathy characterized by sensory ataxia. The neurophysiological features are compatible with length‐dependent demyelination [4, 5].

MAG is a transmembrane glycoprotein that plays a fundamental role in the formation and maintenance of the myelin sheath. Segmental demyelination with deposits of IgM and complement in the myelin sheaths has been demonstrated in anti‐MAG polyneuropathy [5–8].

Most studies reporting on the nature of the anti‐MAG monoclonal IgM have stated that the IgM is almost exclusively kappa‐restricted, but there is only one report addressing IGHV gene usage [9–11]. Substantial clonal and oligoclonal expansions within the IgM memory B‐cell compartment were reported, and 21.5% of single‐sorted cells had identical immunoglobulin heavy (IGH) CDR3 gene sequences [10]. Lee et al. [12] suggested several years ago that the IgM‐secreting clonal B cell is CD5+ showing resemblance to chronic lymphocytic leukemia cells. However, more recent studies have disclosed MYD88 mutations in many patients with anti‐MAG polyneuropathy, suggesting that the clonal B cell is a lymphoplasmacytic‐like B cell [10, 13].

Clinical studies do not provide solid evidence to support B‐cell‐directed treatment for anti‐MAG associated polyneuropathy [14–19]. Two small placebo‐controlled studies did not disclose a significant effect of rituximab following intention‐to‐treat analyses, but per‐protocol analyses indicated that some might benefit from rituximab [17, 18]. Nevertheless, B‐cell‐directed treatment with the monoclonal anti‐CD20 antibody rituximab has become the preferred first‐line treatment as suggested by large uncontrolled observational studies [20, 21] and reviews [5, 22–25]. However, response rates to rituximab monotherapy are low, and response durations are short, as reported in uncontrolled [14–16, 19] and placebo‐controlled studies [17, 18]. The addition of fludarabine, fludarabine+cyclophosphamide, or bendamustine to rituximab has been reported to result in higher and more durable responses according to two uncontrolled studies [26, 27]. Although hematological response has guided the intensity, duration, and choice of treatment in various diseases with monoclonal gammopathies, little attention to B‐cell characteristics and reduction of the M‐component has been made in the studies of anti‐MAG polyneuropathy [21].

The aim of this work was to characterize monoclonal IgM and clonal B cells in patients with anti‐MAG polyneuropathy. Characterization of the clonal B cells in detail may help refine B‐cell‐directed therapy and thus improve treatment efficacy.

## 2. Methods and Patients

### 2.1. Study Design

This is mainly a retrospective cohort study of patients who tested positive for an anti‐MAG antibody assay. Seven patients have been recruited prospectively after we initiated this study in October 2022. The patients still alive provided written informed consent, and the study was approved by the Regional Committee for Medical and Health Research Ethics Southeast Norway (Ref. Number: 490896) and the Data Protection Supervisor, Oslo University Hospital.

### 2.2. Antibody Assessment

Patient sera were screened for anti‐MAG antibodies by a quantitative ELISA assay (Bühlmann Laboratories AG, Schönenbuch, Switzerland). Patient sera with an anti‐MAG antibody titer of 1000 Bühlmann Titer Units (BTU) were considered positive. Sera, which tested positive by the ELISA assay, were also tested by an indirect immunofluorescence assay (Euroimmun, Lübeck, Germany). Only patient sera, which tested positive in both assays, were accepted as positive for anti‐MAG antibodies.

### 2.3. Patient Population

Patients with a monoclonal gammopathy of IgM type who tested positive for anti‐MAG antibodies were identified from the database at the Department of Immunology and Transfusion Medicine, Oslo University Hospital. By August 2024, 54 patients with an anti‐MAG antibody titer above 1000 BTU were identified. Antibody titers between 1000 and 7000 BTU are considered weakly positive with low specificity, whereas titers between 7000 and ‐70000 BTU and above 70000 BTU are considered positive and strongly positive with high specificity for anti‐MAG antibody‐positive polyneuropathy [11]. Patients with antibody titers below 7000 BTU were excluded. Three patients were excluded because confirmatory clinical and neurophysiological data were not available, leaving 42 patients for further investigations (Table 1).

**Table 1:** Patient characteristics.

Patient characteristics	Sex (M/F)	Age at diagnosis	M‐component	Total IgM (g/L)	M‐component (g/L)	Anti‐MAG antibody titer BTU
MAG001	F	59	IgM kappa	3.3	1.0	>70000
MAG002	M	68	IgM kappa	2.6	1.0	>70000
MAG003	M	73	IgM kappa	1.0	0.3	52000
MAG004	F	82	IgM kappa	na	3.0	>70000
MAG006	M	67	IgM kappa	na	4.2	55954
MAG007	M	71	IgM kappa	na	2.0	>70000
MAG008	M	47	IgM kappa	11.1	4.5	>70000
MAG012	M	48	IgM kappa	5.7	1.9	>70000
MAG013	M	70	IgM lambda	11	4.5	10321
MAG014	F	85	IgM kappa	na	1.0	32351
MAG015	F	69	IgM kappa	4.4	1.5	10321
MAG016	M	74	IgM kappa	4.7	2.0	>70000
MAG019	F	72	IgM kappa	6.5	2.0	>70000
MAG020	F	60	IgM kappa	1.6	0.4	24338
MAG021	F	73	IgM kappa	7.6	3.0	>70000
MAG022	M	60	IgM lambda	na	5.0	10108
MAG023	M	56	IgM kappa	na	3.0	53152
MAG024	M	65	IgM kappa	4.7	1.7	>70000
MAG025	F	75	IgM kappa	5.6	1.5	22279
MAG027	M	69	IgM kappa	na	0.4	>70000
MAG028	F	68	IgM kappa	7.2	4.0	>70000
MAG029	F	53	IgM kappa	1.9	0.5	10802
MAG030	M	55	IgM kappa	na	17.1	7734
MAG031	M	76	IgM kappa	na	2.0	41609
MAG032	M	71	IgM kappa	na	12.0	>70000
MAG036	M	63	IgM kappa	na	2.8	>70000
MAG038	M	75	IgM kappa	37.0	19.0	18980
MAG039	F	59	IgM kappa	11.0	6.4	>70000
MAG040	M	73	IgM lambda	na	1.0	>70000
MAG041	M	68	IgM kappa	na	28.0	>70000
MAG042	M	72	IgM lambda	6.1	2.0	>70000
MAG043	M	81	IgM kappa	3.6	2.0	>70000
MAG044	F	53	IgM kappa	2.8	1.4	>70000
MAG045	M	51	IgM kappa	na	7.0	>70000
MAG046	M	50	IgM kappa	12.1	6.0	>70000
MAG047	F	68	IgM kappa	na	0.8	>70000
MAG048	F	68	IgM kappa	4.9	1.0	>70000
MAG049	M	78	IgM kappa	8.5	5.0	18175
MAG050	F	81	IgM kappa	na	5.0	24658
MAG051	F	77	IgM lambda	2.2	0.7	>70000
MAG054	F	81	IgM kappa	5.8	1.6	>70000

Abbreviation: na, not available.

Patients’ medical records were assessed to make sure that patients with a positive test for anti‐MAG antibodies also suffered from polyneuropathy with characteristic clinical and neurophysiological findings. This was done by two experienced neurologists (THP and AHM). No patients were excluded following a review of the medical records.

### 2.4. Flow Cytometry

Flow cytometry on bone marrow samples was done in 31 patients. Available flow cytometric files were reviewed and reassessed by an experienced investigator (LTNO). Based on the markers disclosed by these data files, a standardized protocol for prospective analysis was developed. Data from patients recruited prospectively using this protocol were procured from seven patients.

Flow cytometry analysis was performed as described before [28]. In short, viable cells were gated using the forward scatter (FSC) vs. side scatter (SSC) dot plot of FCS area vs. FCS height. Then, CD45 bright, low SSC events (i.e., lymphocytes) were selected. CD19+ B lymphocytes were gated as CD19‐positive events, negative for T‐lymphocyte and NK‐cell markers. Finally, monoclonal B lymphocytes were separated from the polyclonal B lymphocytes using the immunoglobulin light chain gate, taking advantage of B lymphocytes showing either kappa or lambda light chain restriction in combination with an aberrant phenotype when possible. CR1 (CD35) expression was assessed separately in kappa‐ and lambda‐restricted B lymphocytes. CD13 expression on B lymphocytes was assessed (CD13‐PE, clone 3G8, Becton Dickinson, NJ, USA).

### 2.5. Trephine Biopsies

Trephine biopsies were available in 36 patients, and the biopsies were reassessed by two experienced hematopathologists (SS and UR). MYD88^L265P^ mutational analysis (NM 002468) was performed using a PCR and a SNaPshot multiplex kit (Applied Biosystems). PCR primers and conditions are described elsewhere [29]. The sensitivity of the MYD88^L265P^ mutation analysis was 3%.

### 2.6. Clonality Analysis

Clonality of the IGH chain gene was assessed in two fresh bone marrow samples using the IdentiClone IGH Gene Clonality Assay – ABI Fluorescence Detection Kit (Invivoscribe Technologies). Prior to clonality testing, clonal B cells were isolated by flow cytometry, as previously described [28]. B cells expressing immunoglobulin kappa light chains (IGK^+^) were further subdivided based on the expression of complement receptor 1 (CR1), resulting in two distinct populations: IGK^+^ CR1^−^/low and IGK^+^ CR1^+^. However, the immunoglobulin lambda light chain–expressing (IGL^+^) population was analyzed as a single group without further subdivision. IGH clonality analysis was performed on four populations: total B cells, IGK^+^ CR1^−^/low B cells, IGK^+^ CR1^+^ B cells, and IGL^+^ B cells.

## 3. Results

### 3.1. Patient Characteristics

The patient characteristics are shown in Table 1. Altogether 42 patients (35 retrospectively and 7 prospectively) were included in the study: 17 females and 25 males (sex ratio 0,68) with a median age of 68,5 years (range 47–85 years).

### 3.2. Monoclonal Gammopathy

The monoclonal gammopathy was IgM and ranged from 0.3–28 g/L (median 2 g/L). Light‐chain restriction was kappa in 36 patients and lambda in six patients. We further addressed features of the monoclonal IgM in those with anti‐MAG antibody titers above and below 70 000 BTU. The monoclonal IgM ranged from 0.4–28 g/L (median 2 g/L) in the high‐titer group and 0.3–17.1 g/L (median 2,5 g/L) in the low‐titer group. Three patients with lambda restriction were in the high‐titer group. The male/female ratio was the same for both groups. The results are summarized in Table 1.

### 3.3. Flow Cytometry

**Figure 1:**
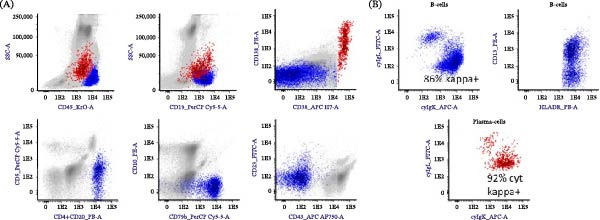
Flow cytometry plots illustrate the typical phenotype for B lymphocytes and plasma cells in bone marrow from a patient with a high‐titer anti‐MAG. Gating strategy: Viable cells were gated using forward scatter (FSC) and side scatter (SSC) parameters, excluding doublets, aggregates, platelets and debris. Mature B cells (blue) were gated as viable singlets and CD45‐bright and CD19+ cells. Plasma cells (red) were gated as viable singlets and CD138+ and CD38+ cells. Clonal B lymphocytes and plasma cells were identified using the immunoglobulin light chain gate. The gating strategy is detailed in Supporting Information 2: Figure S1. Flow data were available on file from 31 patients, and flow data were procured prospectively from 5 patients. (A) B cells were negative for CD5 and CD10 with normal expression of CD45, CD19, CD20, CD79b, CD23, and CD43. Plasma cells identified as CD38+/CD138+ had normal expressions of CD19 and CD45, with no aberrant expression of CD56 (not shown). (B) In B lymphocytes and plasma cells a clear skewness toward kappa expression was observed. Most B lymphocytes showed an aberrant expression of CD13.

**Figure 2:**
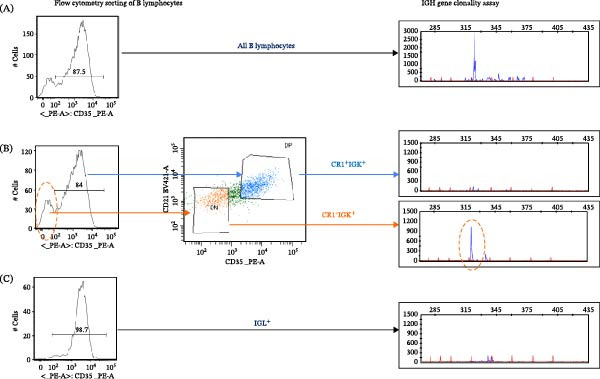
CD35 (complement receptor 1, CR1) expression by clonal B lymphocytes. B lymphocytes were identified as CD45‐bright cells with low SSC positive for CD19 and negative for T‐lymphocyte and NK‐cell markers by flow cytometry. All B lymphocytes are displayed in a histogram (A) showing that most B lymphocytes (87.5%) express high levels of CD35. The IGH gene clonality assay to the right shows that the B lymphocytes include a clonal population. (B) Kappa‐restricted B lymphocytes are displayed in a histogram showing two populations separated by expression of CD35. The IGH gene clonality assay clearly shows that the clonal B lymphocytes are contained in the CD35 low‐kappa‐restricted population. (C) Lambda‐restricted B lymphocytes are displayed in a histogram showing that none of the lambda‐restricted are low in CD35 expression, and the IGH gene clonality assay shows that the lambda‐restricted B lymphocytes do not include clonal B lymphocytes.

Flow cytometry of bone marrow aspirates was performed in 38 patients. The reassessment of the flow cytometry files (31 patients) together with the analyses of fresh samples (seven patients) are summarized in Supporting Information 1: Table S1. These data, together with assessment of fresh samples, indicate the following immunophenotype of the monoclonal B‐cell population: CD5 −CD10 − CD11b−CD13 + CD19 + CD20 + CD23 − CD38 − CD43 − CD44 + CD79b + CD138 − CD200+/(+)light chain bright. Furthermore, we analyzed the flow cytometric records to assess whether the terminal differentiation of the clonal B cells into plasma cells was evident in any of the patient samples. These analyses disclosed that terminal differentiation to plasma cells, as indicated by the expression of CD38/CD138, was disclosed in 12 out of 17 patient samples (Figure 1 and Supporting Information 1: Table S1). We have previously shown downregulation of surface expression of CR1 (CD35) on clonal B lymphocytes in cold agglutinin disease (CAD) [28]. In samples from the patients recruited prospectively, CD35 expression was downregulated exclusively by the clonal B lymphocytes, while CD35 expression on nonclonal B lymphocytes was normal in anti‐MAG polyneuropathy (Figure 2).

### 3.4. Bone Marrow Histology

**Figure 3:**
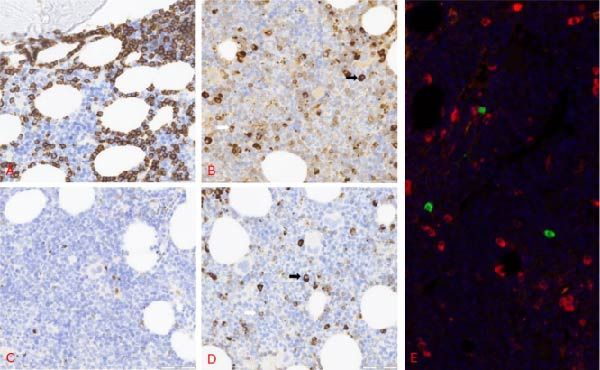
Typical findings in lymphoplasmacytic lymphoma (MAG045). (A) CD20 highlights interstitial and paratrabecular infiltration of small B lymphocytes. (B) Kappa light chain restriction is present with strong expression in plasmacytoid lymphocytes (white arrow) and plasma cells (black arrow) and a weak expression in the B lymphocytes. (C) The lymphoma cells are negative for lambda light chain expression. (D) Strong expression of IgM expression is shown by plasmacytoid lymphocytes (white arrow) and plasma cells (black arrow) and weak expressions by the B lymphocytes. (E) Immunofluorescence staining for kappa light chain (red) and lambda light chain (green) shows kappa light chain restriction in plasma cells and plasmacytoid lymphocytes.

A trephine biopsy had been performed in 36 patients; in four patients, no bone marrow assessment had been performed, and in two patients, bone marrow assessment was by flow cytometry only. The results are summarized in Supporting Information 1: Table S2. In six patients, no clonal B cells could be detected by either histopathology or by flow cytometry, making a diagnosis of monoclonal gammopathy of neurological significance (MGNS). In 14 patients, a mature B‐cell lymphoma was diagnosed; lymphoplasmacytic lymphoma in 12 patients; and marginal zone lymphoma in two patients (both MYD88^L265P^ negative). In the remaining 18 patients, a diagnosis of IgM monoclonal B lymphocytosis was made, and in 11 of these cases, clonal plasma cells were also disclosed. Trephine biopsies from patients with lymphoplasmacytic lymphoma showed typical findings with combined paratrabecular and interstitial infiltration by small lymphocytes, plasmacytoid cells, and clonal plasma cells (Figure 3). MYD88 mutational analysis was performed in 17 patients; a MYD88^L265P^ mutation was detected in 10 patients (Supporting Information 1: Table S2). One of the MYD88‐negative bone marrow specimens was diagnosed as marginal zone lymphoma, four as monoclonal B lymphocytosis, and one with MGNS. In these five cases, it may have been a false negative result due to a low number of clonal B lymphocytes. Interestingly, MYD88^L265P^ was observed in nine out of 10 cases of lymphoplasmacytic lymphoma in which it was assessed.

### 3.5. Treatment and Clinical and Hematological Response

Twelve patients did not receive any treatment, whereas the remaining 30 patients received treatment with the purpose of modulating the disease. The treatment delivered included plasmapheresis (*n* = 1), intravenous immunoglobulin (*n* = 8), rituximab monotherapy (*n* = 8), chemo immune therapy (bendamustine + rituximab *n* = 11, cyclophosphamide + rituximab *n* = 2, trofosfamide + rituximab *n* = 1, bortezomib + dexamethasone + rituximab *n* = 2, cyclophosphamide + bortezomib + dexamethasone + rituximab *n* = 4, cyclophosphamide + carfilzomib + dexamethasone + rituximab *n* = 1), and Bruton’s tyrosine kinase inhibitors (ibrutinib *n* = 3 and zanubrutinib *n* = 3) (Supporting Information 1: Table S3). The retrospective nature of the study did not allow for a detailed and consistent description of responses to the treatment provided. Nevertheless, some information was extracted from the medical records. Two patients (MAG020 and MAG046) experienced durable complete resolution of neurological symptoms following treatment with cyclophosphamide + bortezomib + dexamethasone + rituximab and zanubrutinib, respectively. Both were treated within 1 year of diagnosis, and a complete and very good partial hematological remission were achieved. Five patients experienced durable improvement of neurological symptoms accompanied by a partial hematological remission following rituximab monotherapy (MAG46), bendamustine + rituximab (MAG12, MAG13, and MAG45), and bortezomib + dexamethasone + rituximab (MAG41). Two patients experienced no further progression of neurological symptoms accompanied by a complete (MAG016, rituximab monotherapy) or partial (MAG028, cyclophosphamide + bortezomib + dexamethasone + rituximab) hematological remission (Supporting Information 1: Table S3). No patient reported improvement of neurological symptoms without a hematological response, and a significant reduction of anti‐MAG titer was observed in all patients in which it was assessed. In 21 patients who received disease‐modulating therapy, no symptomatic improvement was observed. One of six patients who received bortezomib‐based therapy had to stop treatment due to progressive polyneuropathy.

## 4. Discussion

In this study, we confirm that a monoclonal gammopathy of the IgM type is found in all patients with anti‐MAG polyneuropathy. In line with previous studies, we found that the monoclonal IgM in the vast majority (>85%) was kappa‐restricted [23]. The underlying B‐cell population as assessed by trephine biopsy and flow cytometry ranged from undetectable (MGNS) to lymphoplasmacytic lymphoma through IgM monoclonal B lymphocytosis. In two patients, the clonal B‐cell population was identified as marginal zone B‐cell lymphoma.

We ascertained that the clonal B cells in anti‐MAG polyneuropathy are CD5‐CD10‐ lymphoplasmacytic‐like cells in most cases, and, importantly, the clonal B‐cell population in at least half of the patients included a proportion of terminally differentiated plasma cells. Apart from the old report by Lee et al. [12] suggesting that the clonal B lymphocytes expressed a CD5+ CLL‐like immunophenotype, little attention has been paid to the immune phenotype of the clonal B cells in anti‐MAG polyneuropathy. However, recent reports have found the MYD88^L265P^ mutation to be prevalent in anti‐MAG polyneuropathy, alluding to a lymphoplasmacytic‐like B‐cell differentiation [10, 13], although the MYD88^L265P^ mutation may be found in other mature B‐cell malignancies [30]. The MYD88^265P^ mutation was disclosed in 10 of 15 cases in which it was investigated in our cohort. Wild‐type MYD88 was found in two marginal zone lymphoma cases and three cases diagnosed as clonal B‐lymphocytosis.

We found CD13 expression to be a feature of the clonal B lymphocytes in our patients, and CD13 expression has been shown to be a hallmark of plasmacytic differentiation in mature B‐cell malignancies [31, 32]. Furthermore, we found clonal plasma cells to be part of the clonal B‐cell population in several of our patients. This finding has implications for the choice of treatment. Appreciating clonal B lymphocytes as the underlying cause of anti‐MAG polyneuropathy established rituximab as the preferred therapy, as suggested by several reviews [5, 22–25]. Whereas several uncontrolled studies have reported a response to rituximab in some patients, although not the majority [14–16, 19–21, 33, 34], two randomized studies have failed to show benefit from rituximab monotherapy [17, 18]. A meta‐analysis of the two prospective randomized studies indicates that one‐third of the patients may have some benefit from rituximab [35]. A more recent review of 23 publications reporting on monotherapy with rituximab states that neurophysiological improvement was evident in 40% of the patients [24]. Common to all these studies is that they do not report on the hematological response, and they state that the response duration is short.

In paraproteinemia‐associated disorders, organ response is closely correlated with the ability to achieve a sustained reduction in monoclonal immunoglobulin levels. A major limitation of previous studies on anti‐MAG polyneuropathy is the lack of consistent reporting on the hematological response, making it difficult to draw meaningful conclusions regarding treatment responses. In CAD, another autoimmune monoclonal IgM gammopathy disorder, rituximab monotherapy resulted in clinical improvement in 50% of the patients, but the response duration was short due to a transient reduction of IgM levels [36, 37]. In CAD, unlike in anti‐MAG polyneuropathy, there are no morphological features of lymphoplasmacytic lymphoma, and MYD88^265P^ is not detected [29]. Nevertheless, complete hematological remissions were rare following rituximab monotherapy [36, 37]. The final analysis of the iNNOVATE study showed that complete remission and very good partial remission were very rare in Waldenström’s macroglobulinemia following treatment with rituximab monotherapy [38]. Combining rituximab with chemotherapeutic agents in CAD resulted in major improvements in efficacy with a high proportion of complete hematological responses and sustained remissions [39]. These findings underscore the potential of combination therapies as a more effective strategy to achieve durable hematological and clinical responses in anti‐MAG polyneuropathy. Including plasma cell‐directed agents in such combination therapies is most likely mandatory to eradicate long‐lived plasma cells.

The burden of symptoms was obviously considered to be high by patients and their healthcare providers, as nearly 80% of the patients in our cohort had received one or more lines of treatment during the disease course. B‐lymphocyte‐directed treatment was most often used. However, the treatment efficacy was not impressive, and an assessment of the hematological response was rarely performed. In anti‐MAG polyneuropathy, the monoclonal IgM accumulates in the myelin sheet which becomes thickened, and widely spaced myelin is a pathognomonic finding [6, 7]. Effective mobilization of monoclonal IgM deposits likely requires achieving complete hematological remission, as evidenced by lessons from AL amyloidosis and CAD patients [39, 40]. To achieve a complete hematological remission (no monoclonal gammopathy, no anti‐MAG antibodies, and no monoclonal B lymphocytes and plasma cells) in anti‐MAG polyneuropathy, future treatment protocols should include drugs that target both plasma cells and B lymphocytes. The underlying B‐lymphoproliferative disease is obviously an indolent disease, as progression to overt clinical lymphoma is rare [22]. Achieving complete hematological remission will probably turn out to be highly durable, as observed in CAD [39]. A consensus report from the tenth International Workshop for Waldenström macroglobulinemia indicated BTK inhibitors and BCL‐2 antagonists as promising treatment options [41]. Indeed, favorable results have been reported following ibrutinib monotherapy [42, 43], ibrutinib in combination with rituximab [38], venetoclax monotherapy [44], and time‐limited ibrutinib and venetoclax in combination [45]. Although complete remissions have been reported, such deep remissions seem to be very rare (approximately in 1%) [38, 43].

In this study, we show that CR1 is downregulated on clonal B lymphocytes but not on the patient’s normal B lymphocytes. We have previously shown this to be the case in CAD as well, and reduced expression of CR1 and CR2 leads to activation, proliferation, and increased antibody production in these cells [28, 46]. This may be a common feature of mature B‐lymphoproliferative disease, which needs further studies [28, 46–48].

In conclusion, monoclonal gammopathy is almost exclusively of the IgM kappa isotype in anti‐MAG polyneuropathy, and often, but not always, clonal plasma cells may be shown to be part of the IgM‐producing B‐cell population. This may explain why rituximab may be efficacious in some patients with anti‐MAG polyneuropathy but not in most patients and indicates that future B‐cell‐directed treatment protocols should include plasma cell‐directed agents to improve efficacy. Including CD13 and CD35 in the flow cytometry panel may help in diagnosing the B‐cell population in anti‐MAG polyneuropathy and in the assessment of residual disease following treatment.

## Author Contributions

**Geir Erland Tjønnfjord:** conception and design, collection and assembly of data, data analysis and interpretation, manuscript writing, manuscript editing, final approval of manuscript. **Liv Toril N. Osnes, Signe Spetalen, and Ulla Haave:** provision of study materials, collection and assembly of data, data analysis and interpretation, manuscript editing, final approval of manuscript. **Trine H. Popperud and Angelina H. Maniaol:** provision of study materials and patients, collection and assembly of data, data analysis and interpretation, manuscript editing, final approval of manuscript. **Ingunn Østlie:** provision of study materials, collection and assembly of data, manuscript editing, final approval of manuscript. **Livia Bajelan:** provision of study materials, data analysis and interpretation, manuscript editing, final approval of manuscript. **Lars Henrik D. Hamnvik, Kristo Marvyin, Dragana Miskovic, Eirik B. Tjønnfjord, Kristoffer Winje, and Magnus Moksnes:** provision of study materials and patients, collection and assembly of data, manuscript editing, final approval of manuscript. **Tor Henrik A. Tvedt:** collection and assembly of data, data analysis and interpretation, manuscript editing, final approval of manuscript. **Ann Kristin Kvam:** conception and design, data analysis and interpretation, manuscript editing, final approval of manuscript. **Agnieszka Malecka:** conception and design, provision of study materials, collection and assembly of data, data analysis and interpretation, manuscript editing, final approval of manuscript.

## Funding

The authors received no specific funding for this work.

## Disclosure

All authors collected the data, reviewed and interpreted the results, and edited the manuscript.

## Consent

Patients provided written informed consent using protocols approved by the Regional Committee for Medical and Health Research Ethics Southeast Norway (Ref. Number: 490896) and the Data Protection Officer, Oslo University Hospital.

## Conflicts of Interest

The authors declare no conflicts of interest.

## Supporting Information

Additional supporting information can be found online in the Supporting Information section.

## Supporting information


**Supporting Information 1** Table S1: Phenotype of clonal B‐lymphocytes based on flow cytometry and immunohistochemistry. Table S2: Diagnosis of the clonal B‐cell population based on immunohistochemistry and flow cytometry. Table S3: Treatment received and treatment outcome.

**Supporting Information 2** Figure S1: Gating strategy to identify clonal B lymphocytes and plasma cells Gating strategy to identify clonal B lymphocytes and plasma cells.

## Data Availability

Data are available upon request from the authors; please contact gtjonnfj@ous-hf.no.
