# Supplementary material for: Anti‐MAG Polyneuropathy: Characterization of the Monoclonal Gammopathy and Clonal B‐Cell Population
Source: J Immunol Res. 2026 Jul 3;2026:1959235. doi: 10.1155/jimr/1959235 (PMC13329836; doi:10.1155/jimr/1959235)
Supplement: Supplementary file 1 — Supporting Information 1 Table S1: Phenotype of clonal B‐lymphocytes based on flow cytometry and immunohistochemistry. Table S2: Diagnosis of the clonal B‐cell population based on immunohistochemistry and flow cytometry. Table S3: Treatment received and treatment outcome. [file JIMR-2026-1959235-s002.pdf]

Supplementary table 1. Phenotype of clonal B lymphocytes based on flow cytometry and immunohistochemistry

	Clone size %	CD45	CD5	CD10	CD11b	CD13	CD19	CD20	CD23	CD38	CD44	CD79b	CD200	KAPPA	LAMBDA	CD43	Clonal plasma cells
MAG001		+	-	-			+	+	-			+	+	+			X
MAG002	2	+	-/+	-			+	+	-	+		+	+	+			X
MAG003								+						+			
MAG004	3,3	+	-	-	-	+/-	+	+	-	-	+	+ +	+/-	+		-	X
MAG005														+			
MAG006	5	+	-	-	-	+/-	+	+	-	-/+		+	+	+			
MAG007								+									X
MAG008	0,92	+	-		-	-	+	++	-	-		(+)	+	+			
MAG012	2,5		-	-				+					+				X
MAG013	2	+	-	-	-	-	+	+	-	-		+	+		+		X
MAG014								+						+			X
MAG015								+									
MAG016																	
MAG019	0,04	+	+	-			+	+						+			
MAG020																	
MAG021		+	-	-			+	+	-			+	+				X
MAG022	33	+	-	-			+	+	-			+	+/-		+	-	
MAG023	10	+	-	-			+	+	-	+				+			
MAG024	0,54	+	-/+	-	-	-	+	+	-	-	+	+	+/-	+			
MAG025	4	+	-	-	-	+	+	+	-	-		+	+/-	+		-	
MAG027		+	-	-	-	-	+	+	-	-		+	+/-		+		
MAG028	1,4	+	-	-	-	+/-	+	+	-	+		+	+/-	+			
MAG029	1,7	+	-	-	-	+/-	+	+	-/+	-	+	+	+	+			
MAG030	41,5	+	-	-			+	+	-			+	+/-	+			X
MAG031	0,5	+	+	-			+	(+)	-	-		-	++	+		+	X
MAG032	1,5	+	+	-	-	+	+	+	-	-		+	+/-	+			X
MAG036	6,4	+	-	-/(+)	-	+/-	+	+	-	(+)	+	++	+	+			X
MAG038			-	-				+	-	-				+			X
MAG039	5,6	+	-	-		+	+	+	-	-	+	+	+	+			X
MAG040								+									
MAG041	4,5	+	-	-		+	+	+	-	-		+	+	+		(+)	X

MAG042																	
MAG043	3,5	+	-	-		-	+	+	-	-	+	+	+	+		-	X
MAG044																	
MAG045	5,5	+	-	-	-	+/-	+	+	-	(+)	++	+	+	+			X
MAG046	1	+	-	-	-	+	+	+	-	+	++	+	(+)	+			X
MAG047	1	+	-	-			+	+	-	-	+	+	+	+			
MAG048	14	+	-	-		+	+	+	-	+/-	+	+	+	+			X
MAG049	2	+	-	-			+	+	-			+	+/-	+			
MAG050	10	+	-	-			+	+	-	-		++	(+)	+		-	X
MAG051	0,2	+	-	-			(+)	(+)	-			++	+/-		+		X
MAG054	2	+	-	-			(+)	+	-			++	(+)	+			X

Supplementary table 2. Diagnosis of the clonal B cell population based on immunohistochemistry and flow cytometry									
	Paratrabecular infiltration	Intrasinusoidal growth	Intertrabecular nodules	% CD20+ B-cells	Clonal plasma cells	% IgM+ plasma cells	Increased mast cells	$MYD88^{L265P}$	Diagnosis based on biopsy and flow cytometry, if available
MAG001	No	No	Yes	10%	Kappa	10%	No	nd	Lymphoplasmacytic lymphoma
MAG002	No	No	No	3%	No	1%	No	nd	IgM Monoclonal B lymphocytosis
MAG003	No	No	No	5%	No	3%	No	nd	IgM monoclonal gammopathy = MGNS*
MAG004	No	No	Yes	5%	Kappa	3%	Yes	nd	IgM Monoclonal B lymphocytosis and plasma cells
MAG005	No	No	No	3%	No	nd	No	nd	IgM monoclonal gammopathy = MGNS
MAG006	Yes	No	Yes	10%	Kappa	1%	No	Positive	Lymphoplasmacytic lymphoma
MAG007	No	No	No	5%	Kappa	3%	No	nd	IgM Monoclonal plasma cells
MAG008	No	No	No	6%	Kappa	2%	No	nd	IgM Monoclonal B lymphocytosis and plasma cells
MAG012	No	No	Yes	5%	No	<0,5 %	No	nd	IgM monoclonal gammopathy = MGNS
MAG013	No	No	No	3%	Lambda	0,50%	No	nd	IgM Monoclonal B lymphocytosis and plasma cells
MAG014	No	No	No	5%	Kappa	8%	No	nd	IGM Monoclonal B lymphocytosis and plasma cells
MAG015	No	No	Yes	4%	No	0,50%	No	nd	IgM monoclonal gammopathy = MGNS
MAG016									No bone marrow assessment done
MAG019	No	No	Yes	3%	No	<0,5 %	No	nd	IgM Monoclonal B lymphocytosis
MAG020				3-4%	No	nd		nd	IgM monoclonal gammopathy = MGNS
MAG021	Yes	No	Yes	8%	Kappa	2%	No	Negative	IgM Monoclonal B lymphocytosis and plasma cells
MAG022	No	Yes	Yes	30%	No	<0,5 %	No	Negative	Marginal zone lymphoma
MAG023	Yes	No	Yes	10%	Kappa	2%	No	Negative	Lymphoplasmacytic lymphoma
MAG024	No	No	No	3%	No	0,50%	No	nd	IgM Monoclonal B lymphocytosis
MAG025	No	No	Yes	7%	No	<0,5 %	No	Positive	IgM Monoclonal B lymphocytosis
MAG027	No	No	Yes	3%	Lambda	0,50%	No	Negative	IgM Monoclonal B lymphocytosis and plasma cells
MAG028	No	No	Yes	7%	No	<0,5 %	No	nd	IgM Monoclonal B lymphocytosis
MAG029	Yes	No	Yes	15%	Kappa	4%	No	nd	Marginal zone lymphoma
MAG030	Yes	No	Yes	40%	Kappa	0,50%	Yes	nd	Lymphoplasmacytic lymphoma
MAG031	No	No	Yes	5%	No	1%	No	nd	IgM Monoclonal B lymphocytosis and plasma cells

MAG032	Yes	No	Yes	15%	No	2%	No	Positive	Lymphoplasmacytic lymphoma
MAG036	No	No	Yes	20%	Kappa	1%	Yes	Positive	Lymphoplasmacytic lymphoma
MAG038	Yes	No	Yes	60%	Kappa	3%	Yes	Positive	Lymphoplasmacytic lymphoma
MAG039	No	Yes	Yes	10%	Kappa	3%	No	Positive	Lymphoplasmacytic lymphoma
MAG040	No	No	No	3%	No	<0,5 %	No	Negative	IgM monoclonal gammopathy = MGNS
MAG041	Yes	No	Yes	10%	Kappa	5%	No	Positive	Lymphoplasmacytic lymphoma
MAG042									No bone marrow assessment done
MAG043	No	No	No	4%	Kappa	4%	No	nd	IgM Monoclonal B lymphocytosis and plasma cells
MAG044									No bone marrow assessment done
MAG045	Yes	No	Yes	25%	Kappa	3%	Yes, slightly	Positive	Lymphoplasmacytic lymphoma
MAG046	No	No	No	5%	Kappa	0,04%	No	nd	IgM Monoclonal B lymphocytosis and plasma cells
MAG047	No	No	yes	2%	Kappa	2%	No	nd	IgM Monoclonal B lymphocytosis and plasma cells
MAG048	Focal	No	Yes	25%	Kappa	3%	No	Positive	Lymphoplasmacytic lymphoma
MAG049	Focal	No	Yes	10%	No	1%	No	nd	IgM Monoclonal B lymphocytosis
MAG050	No	No	Yes	25%	Kappa	15%	Yes, slightly	Positive	Lymphoplasmacytic lymphoma
MAG051	No	No	yes	5%	No	1%	No	Negative	IgM Monoclonal B lymphocytosis and plasma cells
MAG054	No	No	No	5%	No	1%	No	Negative	IgM Monoclonal B lymphocytosis

Supplementary table 3. Treatment received and treatment outcome							
	Plasmapheresis	IVIG	Rituximab	Chemotherapy	BTKi	Neurological response	Hematological response
MAG001	X					No improvement	Not assessed
		X				No improvement	Not assessed
			X			No improvement	No change in IgM and anti-MAG titer
MAG002		X				Unsustained minor improvement	Not assessed
MAG003		X				No improvement	Not assessed
			X			No improvement	No change in IgM and anti-MAG titer
MAG004			X	Trofos, pred*		No improvement	M-component reduced from 2 g/L to 0 g/L; anti-MAG titer not assessed
MAG005						No treatment	No treatment
MAG006		X				Unsustained minor improvement	Not assessed
			X	Bendamustine		No improvement	M-component reduced from 4 g/L to 2 g/L; anti-MAG titer unchanged
MAG007			X			No improvement	No change in IgM and anti-MAG titer
MAG008			X	Bendamustine		No improvement	M-componet reduced from 5 g/L to 1,6 g/L and anti-MAG from >70 000 to 55 584
MAG008					Ibru	No improvement	No change in IgM and anti-MAG titer
MAG012			X	Bendamustine		Durable neurological improvement	Total IgM reduced from 5,5 g/L to 2,3 g/L; M-component and anti-MAG titer not assessed
MAG013			X	Bendamustine		Durable neurological improvement	Total IgM reduced from 11,1 g/L to 4,1 g/L; M-component and anti-MAG titer not assessed
MAG014		X				No improvement	Not assessed
			X			No improvement	No change in IgM and anti-MAG titer
MAG015						No treatment	
MAG016			X			Stable disease for several years	M-componet reduced from 2 g/L to 0,0 g/L and anti-MAG from >70 000 to 1 090
MAG019						No treatment	
MAG020			X	CBD		Complete neurological remission	M-component reduced from 0,5 g/L to 0,0 g/L and anti-MAG titer from 23 233 to undectable
MAG021		X				No benefit	Not assessed
MAG022		X				No improvement	Not assessed
			X	Bendamustine		No improvement	M-component unchanged, anti-MAG titer not assessed
MAG023			X	Cyclophos, pred		No improvement	M-componet not assessed, anti-MAG titer unchanged
			X	Bendamustine		No improvement	M-componet not assessed, anti-MAG titer unchanged
MAG024			X	CBD		Progression	M-component reduced from 1,7 g/L to 0,6 g/L and anti-MAG titer from >70 000 to 63 137
MAG025						No treatment	
MAG027			X			No improvement	No change in IgM and anti-MAG titer
			X	Bendamustine		No improvement	No change in IgM and anti-MAG titer
MAG028			X	CBD		Stable disease for several years	M-component reduced from 3,4 g/L to 0,5 g/L and anti-MAG titer from >70 000 to 55 926

MAG029						No treatment	
MAG030						No treatment	
MAG031			X			No improvement	No change in IgM and anti-MAG titer
			X	CBD		Progression	No change in IgM and anti-MAG titer
MAG032			X	Bendamustine		No improvement	Total IgM reduced from 17,2 g/L to 2,8 g/L; M-component and anti-MAG titer not assessed
MAG036			X	Bendamustine		No improvement	M-component unchanged, anti-MAG titer not assessed; treamtent related death
MAG038					Ibru	No improvement	M-component reduced from 22 g/L to 7 g/L, anti-MAG titer not assessed
MAG039			X	Bendamustine		No improvement	M-component reduced from 11 g/L to 4,5 g/L, anti-MAG titer unchanged
					Ibru	No improvement	No change in IgM and anti-MAG titer
			X	BD		Progression of neuropathy	M-component reduced from 6 g/L to 3,5 g/L, anti-MAG titer unchanged
				CCD		No improvement	M-component reduced from 4,5 g/L to 2,5 g/L, anti-MAG titer unchanged
				Carfil,isatux		No improvement	No change in IgM and anti-MAG titer
MAG040						No treatment	
MAG041			X	BD		Durable neurological improvement	M-component reduced from 28,9 g/L to 9,1 g/L, anti-MAG titer not assessed
MAG042						No treatment	
MAG043			X	Cyclophosph		No improvement	No change in IgM and anti-MAG titer
MAG044						No treatment	
MAG045			X	Bendamustine		Durable neulogical improvement	M-component reduced from 7 g/L to 1 g/L, anti-MAG titer from >70 000 to 30476
MAG046					Zanu	Neurological remission	M-component reduced from 6 g/L to 1 g/L, anti-MAG titer from >70 000 to 25761
MAG047					Zanu	Clinical improvement	M-component reduced from 1,0 g/L to 0,1 g/L, anti-MAG from >70 000 to 24018
MAG048					Zanu	Clinical improvement	M-component reduced from 10 g/L to 3 g/L, anti-MAG iter from >70 000 to 5123
MAG049		X				No improvement	Not assessed
			X			Durable neurological improvement	M-component reduced from 1,8 g/L to 0,3 g/L, anti-MAG titer from 18175 to 6509
MAG050						No treatment	
MAG051						No treatment	
MAG054						No treatment	

* Treatment for synchronous cutaneous DLBCL

CBD=cyclophospamide, bortezomib, dexamethasone; CCD=cyclophosphamide, carfilzomib, dexamethasone

BD=bortezomib, dexamathasone
