# Supplementary figures and images for: Anti‐MAG Polyneuropathy: Characterization of the Monoclonal Gammopathy and Clonal B‐Cell Population
Source: J Immunol Res. 2026 Jul 3;2026:1959235. doi: 10.1155/jimr/1959235 (PMC13329836; doi:10.1155/jimr/1959235)

Supplementary figure 1. Gating strategy to identify clonal B lymphocytes and plasma cells

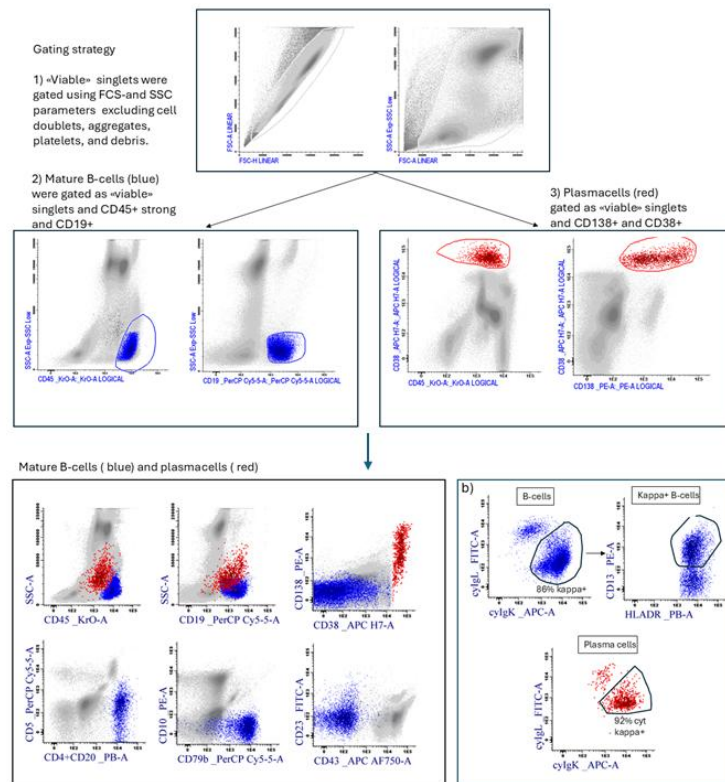

Supplement: Supplementary file 2 — Supporting Information 2 Figure S1: Gating strategy to identify clonal B lymphocytes and plasma cells Gating strategy to identify clonal B lymphocytes and plasma cells. [file JIMR-2026-1959235-s001.pdf]
